# Type-D Personality as a Predictor of Postoperative Pain, Recovery, and Clinical Outcomes After Spine Surgery: Implications for Chronic Pain Management

**DOI:** 10.3390/healthcare13222909

**Published:** 2025-11-14

**Authors:** Christian Riediger, Mark Ferl, Christoph H. Lohmann, Maria Schönrogge, Agnieszka Halm-Pozniak

**Affiliations:** Department of Orthopaedics, University Hospital Magdeburg, Otto von Guericke University Magdeburg, 39120 Magdeburg, Germany; mark.ferl@med.ovgu.de (M.F.); christoph.lohmann@med.ovgu.de (C.H.L.); maria.schoenrogge@med.ovgu.de (M.S.); agnieszka.halm-pozniak@med.ovgu.de (A.H.-P.)

**Keywords:** personality, pain management, spine surgery, postoperative pain, chronic pain

## Abstract

Objectives: To investigate the association between Type-D personality and pain-related outcomes in patients undergoing spine surgery, and to discuss implications for the management of chronic pain conditions. Methods: A prospective cohort of 200 patients scheduled for elective spine surgery was assessed for Type-D personality using the DS14 scale. Postoperative outcomes including pain intensity (VAS), functional recovery (ODI), complication rates, and patient satisfaction (PSI) were measured preoperatively and at 3, 6, and 12 months. Multivariate regression analyses adjusted for age, sex, surgical approach, comorbidities, and baseline health status. Results: Type-D personality was identified in 30% of patients. These individuals reported significantly higher postoperative pain, slower functional recovery, higher complication rates, and lower overall satisfaction compared to non-Type-D patients. Compared with non-Type-D patients, Type-D patients reported higher pain and slower functional recovery at 12 months (VAS β = 0.34, 95% CI 0.18–0.52, *p* = 0.004, Cohen’s d = 0.61; ODI β = 0.31, 95% CI 0.12–0.48, *p* = 0.006, d = 0.58), and lower satisfaction (PSI β = −0.36, 95% CI −0.49 to −0.20, *p* < 0.001, d = 0.66). Conclusions: Type-D personality is associated with worse postoperative pain and recovery. Preoperative psychological assessment and tailored interventions may improve outcomes. These findings highlight the importance of integrating psychosocial screening into pain management strategies for both spine surgery and chronic pain populations.

## 1. Introduction

Chronic pain is a leading cause of disability worldwide and represents a significant clinical challenge in the surgical population. Psychological and behavioral factors strongly influence recovery following surgery. In spine surgery, psychosocial elements often determine the transition from acute to chronic pain and can affect both functional outcomes and satisfaction.

### 1.1. Type-D Personality

Personality traits, particularly Type-D personality (characterized by high negative affectivity and social inhibition), have been shown to predict adverse outcomes in cardiovascular, orthopedic, and pain-related conditions [1,2,3].

Type-D personality, characterized by high levels of negative affectivity (NA) and social inhibition (SI), has been identified as a significant predictor of adverse health outcomes in various medical conditions [2,3]. The term “Type-D” stands for “distressed,” reflecting the chronic negative emotions and tendency to avoid social interactions. Research has shown that individuals with Type-D personality are more likely to experience poor outcomes in cardiovascular diseases, chronic pain conditions, and overall quality of life [1,2,3].

### 1.2. Relevance to Spine Surgery

Spine surgery patients commonly exhibit biopsychosocial interactions between anatomical pathology and psychological vulnerability [4,5]. Despite this, the specific influence of Type-D personality on postoperative recovery after spine surgery remains poorly defined.

### 1.3. Study Objective and Hypothesis

The objective of this study was to investigate the association between Type-D personality and postoperative outcomes in patients undergoing spine surgery. In particular, we examined whether individuals with Type-D personality experience higher levels of postoperative pain, slower functional recovery, a greater incidence of postoperative complications, and lower overall patient satisfaction compared to patients without this personality type.

We hypothesized that the presence of a Type-D personality would negatively influence several aspects of postoperative recovery. Specifically, we expected that Type-D patients would report greater pain intensity, demonstrate less improvement in functional ability, as measured by the Oswestry Disability Index (ODI), and experience a higher rate of postoperative complications. Furthermore, we anticipated that these patients would express lower overall satisfaction with their surgical outcomes and recovery process.

## 2. Materials and Methods

### 2.1. Participants

This prospective cohort study was conducted at the Department of Orthopedics, University Hospital Magdeburg, between March 2022 and December 2024. Adults aged 18–75 years scheduled for elective surgery for degenerative spine conditions were eligible. Exclusion criteria were previous spine surgery, psychiatric disorders other than Type-D, and patients with major comorbidities that could substantially influence pain perception or functional outcomes, including severe cardiovascular disease (NYHA III–IV heart failure), insulin-dependent diabetes with neuropathy, chronic kidney disease (stage ≥ 4), neurodegenerative disorders (e.g., multiple sclerosis and Parkinson’s disease), and prior cerebrovascular events with residual deficits.

### 2.2. Assessment of Type-D Personality

Type-D personality was assessed preoperatively using the 14-item Type-D Scale (DS14) [1], which measures negative affectivity and social inhibition. Patients scoring ≥ 10 on both subscales were classified as Type-D.

### 2.3. Postoperative Outcomes

Pain, function, and satisfaction were assessed using validated, widely applied instruments suitable for postoperative outcome evaluations in spine surgery.

Pain intensity was measured using the Visual Analog Scale (VAS, 0–10 cm), where 0 represents “no pain” and 10 “worst imaginable pain” [6].

Functional disability was evaluated using the Oswestry Disability Index (ODI, 0–100), which quantifies limitations in activities of daily living related to spinal disorders, with higher scores indicating greater impairment [7].

Patient-reported satisfaction with surgical outcome was measured using the Patient Satisfaction Index (PSI, 1–5), capturing overall satisfaction, perceived improvement, and willingness to undergo the same procedure again, with higher values reflecting greater satisfaction [8]. All instruments have demonstrated strong reliability, validity, and sensitivity to clinical change in orthopedic and spine-surgery populations. All evaluations were performed by trained research assistants blinded to surgical allocation.

### 2.4. Statistical Analysis

Data were analyzed using SPSS version 25.0 (IBM Corp. IBM SPSS Statistics for Windows, Version 25.0; IBM Corp.: Armonk, NY, USA, 2017). Continuous variables were compared using *t*-tests and categorical variables were analyzed with chi-square tests. Power Analysis: sample size was based on Vogel et al. (2019) [9], reporting an effect size of f = 0.4 (α = 0.05, power = 0.8). A minimum of n = 180 participants was required. Baseline pain (VAS) and ODI were recorded preoperatively. Multivariate regression adjusted for age, sex, and baseline health status. Multivariate linear regression models were used to examine the association between Type-D status and postoperative outcomes (VAS, ODI, and PSI), adjusting for age, sex, type of surgery (open vs. minimally invasive), comorbidities, and baseline health status. A post hoc power analysis (effect size f = 0.4, α = 0.05, power = 0.8) indicated that a sample size of n = 200 was adequate to detect clinically meaningful differences. Missing data accounted for less than 3% of all observations and were handled using pairwise deletion to maximize the available information for each analysis. According to Little’s MCAR test (*p* > 0.05), missing values were completely random, indicating no systematic bias in the data. Exact *p*-values, 95% confidence intervals (CI), and effect sizes (Cohen’s d) were reported.

### 2.5. Ethical Approval

The study was conducted in accordance with the Declaration of Helsinki and received approval from the Ethics Committee of the Otto-von-Guericke University Magdeburg, Germany (approval numbers 30/22 and 172/15). Written informed consent was obtained from all participants prior to enrollment.

## 3. Results

### 3.1. Patient Characteristics

**Figure 1:**
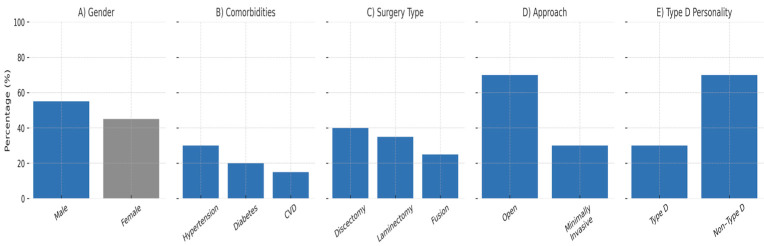
Patient demographics and clinical characteristics. (**A**) Gender distribution (55% male, 45% female). (**B**) Comorbidities including hypertension (30%), diabetes (20%), and cardiovascular disease (15%). (**C**) Type of surgery: discectomy (40%), laminectomy (35%), and spinal fusion (25%). (**D**) Surgical approach: open surgery (70%) vs. minimally invasive (30%). (**E**) Prevalence of Type-D personality (30%) among 200 patients. All panels display percentage (%) on the Y-axis and use a standardized blue-gray MDPI color scheme.

Of the 200 patients included in this study, 60 (30%) were classified as having a Type-D personality. There were no significant differences in baseline demographic or clinical characteristics between the Type-D and non-Type-D groups (Figure 1).

The overall cohort consisted of 200 patients aged 18–75 years (mean ± SD: 50.5 ± 12.4 years), with 55% male and 45% female participants. The most common comorbidities were hypertension (30%), diabetes mellitus (20%), and cardiovascular disease (15%).

Regarding surgical characteristics, 40% of patients underwent discectomy, 35% underwent laminectomy, and 25% underwent spinal fusion procedures. The majority of surgeries (70%) were performed using an open approach, while 30% were conducted using minimally invasive techniques.

### 3.2. Pain and Functional Recovery

Preoperative baseline pain (VAS) was recorded for all patients to allow for a longitudinal comparison. At baseline, patients with a Type-D personality reported slightly higher pain and disability scores compared to non-Type-D patients (VAS: 6.5 vs. 5.5; ODI: 40 vs. 35). Postoperatively, Type-D patients experienced persistently higher pain intensity and slower functional recovery across all follow-up periods. At 12 months, the mean VAS score was 4.0 in Type-D patients versus 2.0 in non-Type-D patients (*p* < 0.01). Functional disability also remained greater in the Type-D group with ODI scores of 30 versus 10 at 12 months (*p* < 0.01).

**Figure 2:**
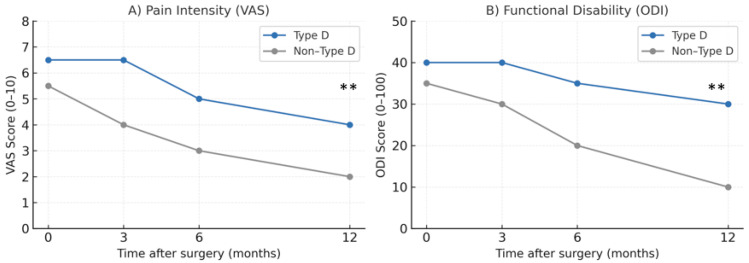
Pain intensity and functional disability over 12 months after spine surgery. (**A**) Pain levels measured by the Visual Analog Scale (VAS, 0–10). (**B**) Functional disability measured by the Oswestry Disability Index (ODI, 0–100). The blue line represents Type-D patients, and the gray line represents Non-Type-D patients. ** indicates *p* < 0.01 for Type-D vs. Non-Type-D at 12 months (two-tailed *t*-test). ** *p* < 0.01.

Longitudinal analyses revealed significant differences in postoperative pain and functional recovery trajectories between the two groups (Table 1 and Table 2). Type-D patients consistently demonstrated higher mean VAS scores, with slower pain reduction compared to non-Type-D patients. Likewise, ODI scores reflected delayed improvement in functional capacity among Type-D patients persisting up to 12 months postoperatively (*p* < 0.01). The progression of postoperative pain and disability over time is illustrated in Figure 2.

### 3.3. Complication Rates

**Figure 3:**
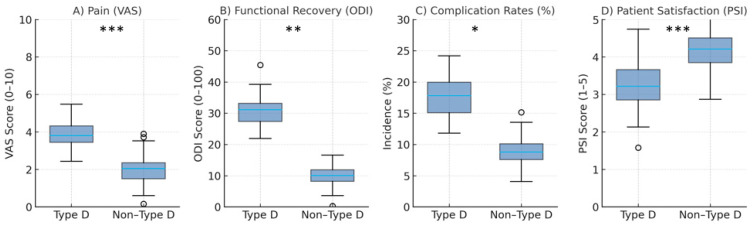
Summary of outcome differences between Type-D and Non-Type-D patients. (**A**) Pain levels (VAS). (**B**) Functional recovery (ODI). (**C**) Complication rates (%). (**D**) Patient satisfaction scores (PSI, 1–5). Boxplots illustrate higher pain and disability, greater complication rates, and lower satisfaction among Type-D patients (all *p* < 0.05). Boxes represent interquartile ranges (IQR), lines indicate medians, and whiskers extend to 1.5 × IQR. Significance markers are shown above each panel (**A**–**D**): * *p* < 0.05, ** *p* < 0.01, *** *p* < 0.001.

Type-D patients exhibited a higher overall incidence of postoperative complications compared with non-Type-D patients (20% ± 5 vs. 10% ± 3, *p* = 0.02). These findings indicate that individuals with Type-D personality traits face a substantially greater risk of postoperative adverse events. The most frequent complications among Type-D patients included superficial (8%) and deep (4%) surgical site infections, delayed wound healing (6%), and transient neurological symptoms such as nerve root pain (5%). Permanent nerve damage was observed in 2% of Type-D patients compared to 1% in the non-Type-D group. Cardiovascular events, including deep vein thrombosis (3%) and pulmonary embolism (1%), as well as respiratory complications such as pneumonia (2%), also occurred more frequently in the Type-D group, although at lower absolute rates. Prolonged hospital stays were recorded in 10% of Type-D patients compared with 5% of non-Type-D patients. Likewise, readmission rates were higher among Type-D patients, both at 30 days (6% vs. 3%) and 90 days postoperatively (10% vs. 5%). These findings are further illustrated in Figure 3, which summarizes the between-group differences in postoperative complications and related outcomes. Overall, the results suggest that Type-D personality is associated with an increased risk of infections, delayed wound healing, and extended hospitalization following spine surgery.

### 3.4. Patient Satisfaction

Overall patient satisfaction was significantly lower among Type-D patients compared with non-Type-D patients (mean PSI: 3.2 ± 0.8 vs. 4.2 ± 0.6, *p* < 0.001), as shown in Table 3. These results demonstrate consistently reduced satisfaction across all assessed domains in patients with Type-D personality traits. While non-Type-D patients generally reported high levels of satisfaction with pain relief, functional recovery, and overall surgical outcome, Type-D patients rated their experiences as only moderate. The difference was most pronounced for satisfaction with pain control and functional improvement, underscoring the influence of psychosocial and personality-related factors on subjective outcome evaluation. These group differences in satisfaction are further illustrated in Figure 3, which highlights the disparity between Type-D and non-Type-D patients across the analyzed satisfaction domains.

A summary of the multivariate regression analyses examining the independent effect of Type-D personality on postoperative outcomes (VAS, ODI, and PSI) is presented in Table 4.

No significant interaction was found between age (<50 vs. ≥50) or surgical approach (open vs. minimally invasive).

## 4. Discussion

This study demonstrates that Type-D personality is associated with less favorable postoperative outcomes in patients undergoing spine surgery. Specifically, individuals with Type-D traits experienced higher postoperative pain, slower functional recovery, and lower overall satisfaction during the 12-month follow-up period. These associations remained significant after adjusting for demographic and surgical covariates, supporting a biopsychosocial contribution to postoperative recovery in spine surgery.

Our findings align with previous reports linking emotional and behavioral traits to surgical recovery and pain chronification [1,2,3,9,10,11,12]. Type-D individuals are characterized by negative affectivity and social inhibition, both of which contribute to maladaptive stress responses and reduced coping ability [13,14]. Physiologically, these traits are associated with enhanced hypothalamic–pituitary–adrenal (HPA) axis activation and sympathetic overactivity, resulting in pro-inflammatory states that may delay tissue repair [15,16]. From a psychological perspective, Type-D individuals often exhibit catastrophizing and avoidant coping strategies, which may exacerbate pain perception and hinder rehabilitation adherence [17,18,19]. Consequently, both neurobiological and behavioral pathways likely mediate the relationship between Type-D personality and postoperative outcomes.

These results highlight the potential value of preoperative psychosocial screening using simple, validated instruments such as the DS14 questionnaire. Identifying high-risk patients allows for early psychological support and better perioperative management. Interventions such as cognitive-behavioral therapy (CBT), stress-management training, or mindfulness-based programs have been shown to reduce negative affectivity and enhance coping [20,21,22]. Integrating such approaches into multidisciplinary spine surgery care could improve recovery trajectories and patient satisfaction. From a practical standpoint, integrating psychological assessment into perioperative care is recommended. Routine screening for Type-D personality may help identify high-risk individuals who could benefit from intensified multimodal pain management, closer follow-up, and structured aftercare programs. Interdisciplinary collaboration among surgeons, psychologists, pain specialists, and rehabilitation experts is essential to translate these insights into clinical practice, consistent with the biopsychosocial model of pain [4].

Finally, while this study strengthens the evidence linking Type-D personality to postoperative outcomes, we acknowledge that other unmeasured psychological and biological factors may also contribute. Our conclusions are therefore framed in associative rather than causal terms. Future prospective studies should validate these findings in larger, multicenter cohorts and evaluate whether psychological interventions targeting Type-D traits can improve recovery and long-term quality of life in surgical patients.

### Limitations of the Study

This study has several limitations that should be acknowledged. First, the cohort was derived from a single tertiary center, which may restrict the generalizability of the findings to other settings. Although the study was adequately powered, larger multicenter trials are warranted to confirm these results in broader surgical populations.

Second, patients with major psychiatric disorders were excluded to isolate the specific influence of Type-D personality traits. While this approach improved internal validity, it may limit the external generalizability of the results, as psychological comorbidities frequently coexist with chronic pain and may interact with Type-D features. Future research should explore these interactions and their combined impact on postoperative outcomes.

Third, the wide age range and heterogeneity in surgical procedures may introduce residual confounding despite adjustment and subgroup analyses. Additionally, the reliance on self-reported instruments (DS14, VAS, ODI, and PSI) could introduce response bias related to subjective perception or recall.

Finally, the 12-month follow-up period, while sufficient to capture short- and mid-term outcomes, may not reflect long-term trajectories of pain or late complications. Extended longitudinal studies are needed to evaluate the durability of these associations over time.

## 5. Conclusions

Type-D personality was associated with higher postoperative pain, slower functional recovery, increased complication rates, and lower satisfaction following spine surgery.

These findings emphasize the need for a biopsychosocial approach to surgical care, integrating psychological assessment and support into preoperative planning.

Further research should explore whether targeted psychosocial interventions can mitigate these risks and improve long-term surgical outcomes.

## Figures and Tables

**Table 1:** Pain levels (VAS) in Type-D and non-Type-D patients.

Timepoint	Type-D (Mean ± SD)	Non-Type-D (Mean ± SD)	Difference/β	95% CI	*p*-Value	Cohen’s d
Preoperative	6.5 ± 1.0	5.5 ± 0.9	β = 0.20	−0.02–0.42	0.074	0.35
3 months	6.5 ± 1.2	4.0 ± 1.0	β = 0.38	0.22–0.55	0.001	0.72
6 months	5.0 ± 1.1	3.0 ± 0.8	β = 0.36	0.19–0.52	0.002	0.66
12 months	4.0 ± 1.0	2.0 ± 0.7	β = 0.34	0.18–0.52	0.004	0.61

Abbreviations: Pain levels (Visual Analog Scale—VAS).

**Table 2:** Functional recovery (ODI) in Type-D and non-Type-D patients.

Timepoint	Type-D (Mean ± SD)	Non-Type-D (Mean ± SD)	Difference/β	95% CI	*p*-Value	Cohen’s d
Preoperative	40 ± 5	35 ± 5	β = 0.15	−0.05–0.34	0.142	0.30
3 months	40 ± 6	30 ± 4	β = 0.33	0.15–0.50	0.003	0.60
6 months	35 ± 5	20 ± 3	β = 0.32	0.14–0.49	0.004	0.59
12 months	30 ± 4	10 ± 2	β = 0.31	0.12–0.48	0.006	0.58

Abbreviations: Functional recovery (Oswestry Disability Index—ODI).

**Table 3:** Patient satisfaction scores (PSI) in Type-D and non-Type-D patients.

Domain	Type-D (Mean ± SD)	Non-Type-D (Mean ± SD)	Difference/β	95% CI	*p*-Value	Cohen’s d
Overall satisfaction	3.2 ± 0.8	4.2 ± 0.6	β = −0.36	−0.49–−0.20	<0.001	0.66
Satisfaction with pain relief	3.0 ± 0.8	4.0 ± 0.7	β = −0.34	−0.47–−0.18	0.001	0.62
Satisfaction with functional recovery	3.1 ± 0.7	4.1 ± 0.6	β = −0.33	−0.46–−0.17	0.002	0.60
Satisfaction with surgical outcome	3.3 ± 0.7	4.3 ± 0.6	β = −0.35	−0.48–−0.19	0.001	0.63
Willingness to undergo surgery again	3.2 ± 0.8	4.2 ± 0.7	β = −0.34	−0.47–−0.18	0.004	0.62
Recommendation to others	3.4 ± 0.8	4.4 ± 0.6	β = −0.35	−0.48–−0.19	0.002	0.63

**Table 4:** Multivariate regression analyses predicting postoperative outcomes. Linear regression results for the association between Type-D personality and outcomes (VAS, ODI, and PSI) at 12 months.

Dependent Variable	β (Type-D)	95% CI	*p*	Adj. *R*^2^	Covariates
Pain (VAS 12 mo)	0.34	0.18–0.52	0.004	0.29	Age, sex, comorbidities, surgery type
Function (ODI 12 mo)	0.31	0.12–0.48	0.006	0.26	Age, sex, comorbidities, surgery type
Satisfaction (PSI 12 mo)	−0.36	−0.49–−0.20	<0.001	0.31	Age, sex, comorbidities, surgery type

## Data Availability

The data supporting the findings of this study are available from the corresponding author upon reasonable request. Due to privacy and ethical restrictions, data are not publicly available.
